# Association of prenatal lipid‐based nutritional supplementation with fetal growth in rural Gambia

**DOI:** 10.1111/mcn.12367

**Published:** 2016-10-02

**Authors:** William Johnson, Momodou K. Darboe, Fatou Sosseh, Patrick Nshe, Andrew M. Prentice, Sophie E. Moore

**Affiliations:** ^1^ MRC Human Nutrition Research Cambridge UK; ^2^ MRC International Nutrition Group MRC Unit The Gambia Fajara, Banjul The Gambia

**Keywords:** fetal growth, gestational weight gain, lipid‐based nutritional supplement, randomized control trial, seasonality, The Gambia

## Abstract

Prenatal supplementation with protein‐energy (PE) and/or multiple‐micronutrients (MMNs) may improve fetal growth, but trials of lipid‐based nutritional supplements (LNSs) have reported inconsistent results. We conducted a post‐hoc analysis of non‐primary outcomes in a trial in Gambia, with the aim to test the associations of LNS with fetal growth and explore how efficacy varies depending on nutritional status. The sample comprised 620 pregnant women in an individually randomized, partially blinded trial with four arms: (a) iron and folic acid (FeFol) tablet (usual care, referent group), (b) MMN tablet, (c) PE LNS, and (d) PE + MMN LNS. Analysis of variance examined unadjusted differences in fetal biometry *z*‐scores at 20 and 30 weeks and neonatal anthropometry *z*‐scores, while regression tested for modification of intervention‐outcome associations by season and maternal height, body mass index, and weight gain. Despite evidence of between‐arm differences in some fetal biometry, *z*‐scores at birth were not greater in the intervention arms than the FeFol arm (e.g., birth weight *z*‐scores: FeFol −0.71, MMN −0.63, PE −0.64, PE + MMN −0.62; group‐wise *p* = .796). In regression analyses, intervention associations with birth weight and head circumference were modified by maternal weight gain between booking and 30 weeks gestation (e.g., PE + MMN associations with birth weight were +0.462 *z*‐scores (95% CI [0.097, 0.826]) in the highest quartile of weight gain but –0.099 *z*‐scores (−0.459, 0.260) in the lowest). In conclusion, we found no strong evidence that a prenatal LNS intervention was associated with better fetal growth in the whole sample.

## 1 INTRODUCTION

In the 2013 Maternal and Child Nutrition series in the Lancet, Black et al. (2013) estimated that undernutrition in the aggregate was responsible for 45% of child mortality, with fetal growth restriction alone accounting for 12% of deaths. This series also included a comprehensive review of nutritional interventions which concluded, among other things, that balanced prenatal protein‐energy (PE) and multiple‐micronutrient (MMN) supplementation could potentially reduce fetal growth restriction and thus the risk of small‐for‐gestational age (SGA) birth (Bhutta et al., 2013). This finding is in line with the most recent Cochrane reviews (Haider & Bhutta, 2015; Ota, Hori, Mori, Tobe‐Gai, & Farrar, 2015). Given the adverse consequences of SGA for mortality (Katz et al., 2013), and its links through postnatal growth failure with a wide range of health and human capital outcomes (Adair et al., 2013; Christian et al., 2013), there is a clear need to understand which routes of prenatal nutritional supplementation are most effective and in whom.

Recently developed lipid‐based nutritional supplements (LNSs), which are affordable, safe, can be produced locally, and have a long shelf‐life, have been shown to be a very effective option for the community‐based treatment of severe malnutrition if a high dose is given (Briend & Collins, 2010; Tekeste, Wondafrash, Azene, & Deribe, 2012; WHO, 2007). They may also provide a route of MMN delivery that may be more preferable and efficacious than other products. A trial in Ghanaian infants, for example, found that LNS fortified with MMN had a positive effect on some growth and motor development outcomes compared to two other types of MMN supplements (Sprinkles powder and crushable Nutritabs) for home fortification of complementary foods (Adu‐Afarwuah, Lartey, Brown, Zlotkin, & Dewey, 2007). Few trials have given LNS to pregnant women, and their results have been equivocal. One study in Malawi found no strong evidence of an effect on birth size of a small‐quantity‐LNS product (SQ‐LNS 118 kcal/day) fortified with MMN compared to either an iron and folic acid (FeFol) arm or a MMN arm (Ashorn et al., 2015). Whereas, a study in Bangladesh reported significant effects of fortified SQ‐LNS (118 kcal/day) compared to a FeFol arm on a range of birth size outcomes, including stunting (relative risk 0.83; 95% confidence interval (CI) [0.71, 0.97]); this effect of SQ‐LNS on reduced stunting risk was strongest in women aged ≤24 years or with household food insecurity (Mridha et al., 2016). Further, one study in Ghana reported significant effects of fortified SQ‐LNS (118 kcal/day) compared to a MMN only arm on birth weight (+139 g; 6, 272) and birth length (+6.7 mm; 0.6, 12.7) only among the sub‐group of primiparous women (Adu‐Afarwuah et al., 2015), and one study in Burkina Faso reported significant effects of fortified LNS (372 kcal/day) compared to a MMN only arm on birth length (+13.5 mm; 6.5, 20.5) only among birth occurring at the end of the nutritionally debilitating rainy season (Huybregts et al., 2009; Toe et al., 2015). The latter finding is in agreement with previous work from our group in rural Gambia, where we found that a daily high‐energy ground‐nut biscuit supplement providing approximately 1000 kcal/day of energy increased birth weight by 94 g (31, 157) for births occurring in the dry season but by 201 g (132, 270) for births occurring in the rainy season (Ceesay et al., 1997).

It appears that fortified LNS may impact on fetal growth and development most among women who are more nutritionally vulnerable; rural Gambian women represent one such group, especially during the rainy season (Poppitt, Prentice, Goldberg, & Whitehead, 1994; Poppitt, Prentice, Jequier, Schutz, & Whitehead, 1993; Prentice, Whitehead, Roberts, & Paul, 1981; Rayco‐Solon, Fulford, & Prentice, 2005). The existing literature has focused on anthropometry taken at birth as a proxy for total fetal growth, but investigation using fetal biometry measures would provide a more dynamic picture and allow quantification of the ages in development when supplementation might first start to affect growth. The aim of the present study was to conduct a post‐hoc analysis, in a prenatal LNS trial with fetal biometry starting early in gestation (as well as neonatal anthropometry), to test the associations of LNS with fetal growth. Further, we explored how efficacy of the interventions might vary depending on season and, associated to this, mothers' nutritional status.

**Key messages**

- Improving pregnant women's diet with LNS did not significantly increase offspring birth weight, length, and head circumference in rural Gambia in the whole sample.
- In sub‐group analyses, however, positive and significant associations of all interventions with birth weight and head circumference were observed among women who demonstrated the greatest gestational weight gain.
- Further investigation is needed to understand whether or not, which, and how environments conducive to better gestational weight gain allow LNS to be utilized by the mother to support fetal growth in resource poor settings.

## 2 PARTICIPANTS AND METHODS

### 2.1 Sample

The sample comprised 620 mothers and their singleton offspring (304 males; 316 females) enrolled in the early nutrition and immune development trial (ENID; trial registration: ISRCTN49285450) in rural Gambia. As shown in Supplementary Figure 1, the sample for the present paper was selected from the total ENID sample (*N* = 875) based on the offspring being live births with complete birth weight and gestational age data. Defining characteristics were not different between this sample and those ENID participants who did not meet the inclusion criteria (*N* = 875–620 = 255). For example, where data were available, the comparison was 162.0 versus 61.7 cm (*p* = .442) for maternal height at booking, 29.6 versus 29.9 years for maternal age at booking (*p* = .642), and 37.9 versus 38.7 for the percentage of births occurring in the raining season (*p* = .861).

**Figure 1:**
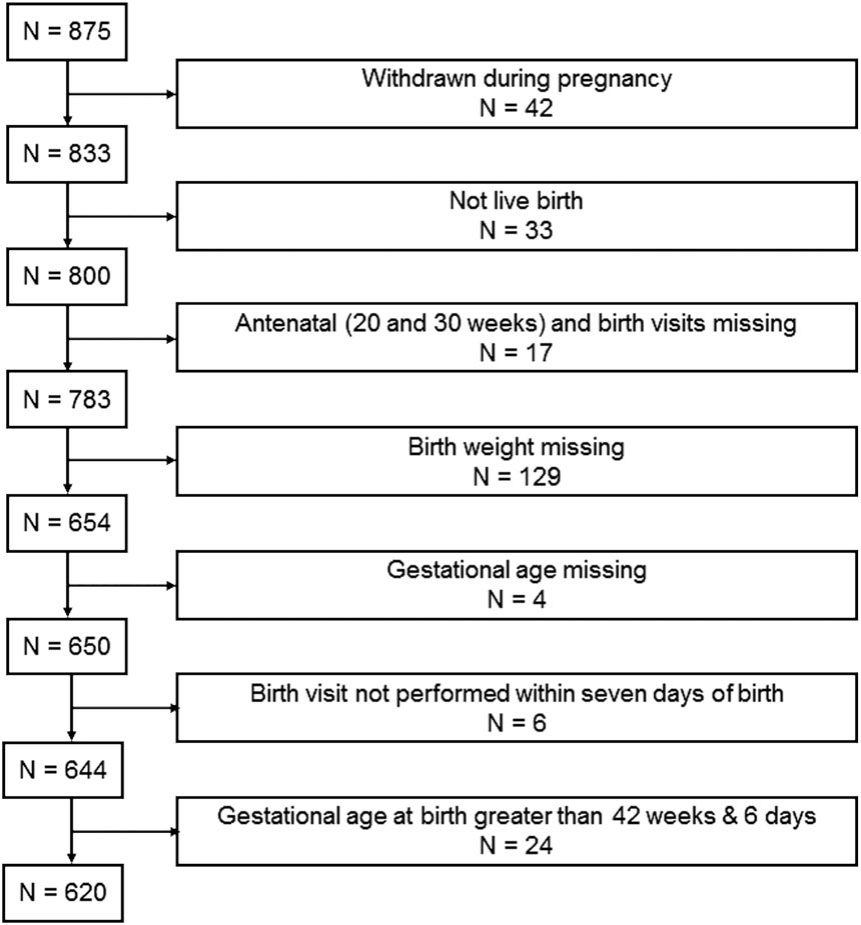
Flow diagram of sample selection

### 2.2 Study design and intervention

The ENID trial has been described in detail elsewhere (Moore et al., 2012), but briefly is a randomized, partially blind trial to assess whether or not nutritional supplementation to pregnant women (from <20 weeks gestation to term) and their infants (from 6 to 12 months of age) can enhance immune development. Pregnancies were identified through monthly surveillance in all eligible non‐pregnant women of reproductive age (18–45 years) in the West Kiang region of The Gambia; date of last menstrual period was assessed, and a urine test was conducted if a menstrual period was missed. Women confirmed by ultrasound as being between 10 and 20 weeks pregnant at a clinic booking visit were randomized to one of four arms: (a) FeFol, (b) MMN, (c) PE, and (d) PE + MMN.

Supplementation commenced the following week, with the first two arms receiving daily tablet supplements and the latter two arms receiving daily LNS. Both supplement types (tablets and LNS) were distributed on a weekly basis to participating women. Women were supplied with 14 tablets per week in individual bottles and advised to take two tablets per day, preferably with food. LNS were supplied in jars, with a single (daily) dose per jar. Women were encouraged to consume the whole jar each day, with the option of eating it straight from the jar (spoons were supplied) or eating it with food. Given the common practice of sharing from a family bowl, women were encouraged to take a separate portion of the food from the family bowl and mix the LNS into that. The first women started to receive supplementation in January 2010, and the final infant was born in February 2014. Compliance was assessed through the collection of all unused supplements at the end of each week. For tablets, a count on remaining tablets was performed, and for LNS products, a score based on the amount of supplement left remaining in the jar was made (empty, half‐empty, and full). A compliance percentage was computed for each woman by dividing the number of LNS pots or tablets the woman consumed by the number she was offered, and multiplying by 100.

The nutritional composition of the intervention products is detailed in the study protocol published by Moore et al. (2012). Briefly, all arms received the Gambian government guidelines for iron (60 mg/day) and folic acid (400 μg/day) supplementation during pregnancy. The two arms also receiving MMN were additionally provided with two times the UNICEF/World Health Organization (WHO)/United Nations University formulation of key micronutrients, and the two arms also receiving PE were additionally provided with 746 kcal/day of energy from protein and lipids.

From 6 to 12 months of age, infants were further randomized to a LNS supplement, with or without additional MMN, but this second randomization stage does not need to be considered for the purposes of the present paper as our focus is on fetal growth.

### 2.3. Ethics

The trial was approved by the joint Gambia Government/MRC Unit, The Gambia Ethics Committee (Project number SCC1126v2). Written informed consent was obtained from all women prior to enrolment into the trial. The trial observed Good Clinical Practice Standards and the current version of the Helsinki Declaration. The trial was registered as ISRCTN49285450.

### 2.4. Measurements

This paper uses data from the clinic enrolment or “booking” visit, subsequent clinic visits at 20 and 30 weeks of gestation, and a home visit performed within 72 hr of birth.

#### 2.4.1. Fetal biometry

At the three prenatal clinic visits, fetal biometry was assessed via ultrasound using a Siemens ACUSON Antares Ultrasound Imaging System (Siemens Medical Solutions United States of America (USA) Inc; California, USA with a CH6–2 (5.71 MHz) transducer). Using the built‐in equations, the purpose of the fetal biometry measurements taken at booking was to estimate gestational age. This estimation was based on crown‐rump length (CRL) if gestational age was <12 weeks, or bi‐parietal diameter (BPD) if CRL was too large to be accurately measured or gestational age was ≥12 weeks. If CRL was not yet measurable, gestational age was estimated according to the size of the gestational sac, and the woman's booking visit was rescheduled for 12 weeks gestation. Subsequently, at the 20 and 30 week visits measurements of femur length (FL), head circumference (HC), occipital‐frontal diameter (OFD), and abdominal circumference (AC), as well at BPD were performed using standard methods (Meire & Farrant, 1995; O'Brien & Queenan, 1981). These measurements were taken on each fetus at each visit by one of two sonographers, who were blind to women's the allocation group. Prior to the start of the study, the two sonographers were trained in fetal biometry, and their measures were standardized according to the protocols as detailed and published by Neufeld, Wagatsuma, Hussain, Begum, and Frongillo (2009). Standardization exercises were performed for each outcome measure at each time point until a level of inter‐ and intra‐observer reliability deemed acceptable by the trainer was reached.

#### 2.4.2. Neonatal anthropometry

Neonatal anthropometry was performed in the infant's home, by the study midwife and within 72 hr of delivery. Weight was measured using digital infant scales (Seca mobile digital babyscale 334; UK) with the infant in minimal clothing and to the nearest 10 g. Length was measured on a portable infant rollameter (Rollameter 100; Harlow Healthcare, UK) to the nearest 0.1 cm. HC was measured using standard circumference tapes (Seca; UK). All measures were made using standard protocols, and equipment was regularly validated.

#### 2.4.3. Maternal factors

Maternal weight and height were measured at the booking and 20 and 30 week visits using standard techniques and equipment (Tanita DH305 scales [Tanita Corporation; Japan] and Leicester height measure [Seca 214; UK]). Maternal date of birth and thus age at booking were ascertained from the West Kiang Demographic Surveillance System (Hennig et al., 2015). Maternal parity was computed, using questionnaire data collected at booking, as the number of deliveries (i.e., alive children, dead children, and still births) excluding abortions.

### 2.5. Statistics

All fetal biometry and maternal and neonatal anthropometry variables were measured in triplicate, and here, we used the median across the three recordings. To account for small differences in timing of assessment and enable comparison across the different measures, fetal biometry were converted to *z*‐scores according to the INTERGROWTH‐21st standard (Papageorghiou et al., 2014), and neonatal anthropometry were converted to *z*‐scores according to the WHO child growth standard (WHO Multicentre Growth Reference,Study Group, 2006). Further, low birth weight was defined as birth weight <2.5 kg, SGA as weight‐for‐gestational age <10th percentile of the INTERGROWTH‐21st standard (Villar et al., 2014), and preterm as a gestational age at birth <37 0/7 weeks. Maternal body mass index (BMI) at booking was computed as weight (kg)/height (m)^2^. Maternal weight gain variables from booking to 20 weeks, booking to 30 weeks, and 20 to 30 weeks, were calculated and expressed as kg per week. Analyses using standardized residual measures that account for regression to the mean produced similar results (Keijzer‐Veen et al., 2005), so we only present results using the simpler and easier to interpret measures. Given the focus of the present paper, season of assessment was not modeled using Fourier terms (Fulford, Rayco‐Solon, & Prentice, 2006) but instead was approximated using binary variables (November to May = dry or June to October = rainy).

Descriptive statistics for baseline variables were produced, stratified by intervention arm. Unadjusted between‐arm differences in primary and secondary outcomes were tested using analysis of variance or Kruskal–Wallis for continuous variables and chi‐squared tests for categorical variables.

General linear regression models were used to test whether or not intervention associations (i.e., MMN, PE, or MMN + PE vs. FeFol) with fetal biometry (FL, HC, BPD, OFD, and AC) and neonatal anthropometry (weight, length, HC, and weight‐for‐length [WFL]) Z‐scores were modified by the following a priori specified variables: season of assessment, maternal height and BMI at booking, and maternal weight gain from booking. These variables were chosen based on evidence from previous publications that fortified LNS may impact on fetal growth and development most among women who are more nutritionally vulnerable (Mridha et al., 2016; Adu‐Afarwuah et al., 2015; Huybregts et al., 2009; Toe et al., 2015). For each combination of the continuous outcomes and the potential modifiers, a model was built including linear intervention‐by‐potential modifier terms. If at least one of the corresponding *p*‐values was <.05 or if there was evidence of association modification for the other dimensions assessed at that visit, subsequent models stratified according to the modifier were built. These stratified models were adjusted for sex, gestational age and season at assessment, maternal height and BMI at booking, and maternal weight gain from booking (i.e., booking to 20 weeks for the 20 week models and booking to 30 weeks for the 30 week and birth models); neonatal anthropometry models additionally included age at assessment and parity. Given our sample size and the hypothesised association modification, logistic models for binary outcomes (e.g., SGA) were not performed because of the reduced power this approach provides.

To investigate if compliance or length of time on supplement were affecting the efficacy of the intervention, sensitivity analyses were performed removing individuals in the bottom quartile of compliance (standardized within each arm) or length of time on supplement. Further, given that the majority of exclusions were due to missing birth weight (*N* = 129), analyses were rerun using neonatal anthropometry from a week one visit instead of the birth visit.

All analyses were performed in Stata 14 (StataCorp LP; College Station, Texas, USA).

## 3 RESULTS

Baseline characteristics of the study sample, stratified according to intervention arm, are shown in Table 1. Maternal variables (e.g., age, weight, height, gestational age, and parity) were similar across the fours arms, but mean fetal BPD was larger in the PE arm (30.2 mm) compared to the other arms (28.5–29.8 mm).

**Table 1:** Baseline characteristics of study sample, by intervention arm

		Tablets		LNS	
		FeFol	MMN	PE	PE + MMN
		*N* = 146	*N* = 164	*N* = 151	*N* = 159
Gestational age (weeks)	Mean (SD)	13.9 (3.4)	13.9 (3.4)	13.8 (3.3)	13.4 (3.2)
Bi‐parietal diameter (mm) (*N* = 194 missing)	Mean (SD)	29.6 (9.5)	29.8 (9.8)	30.2 (9.6)	28.5 (8.9)
Crown‐rump length (mm) (*N* = 426 missing)	Mean (SD)	29.1 (8.9)	31.2 (9.3)	34.8 (11.3)	30.1 (9.3)
Season of measurement					
Nov–May (dry)	*N* (%)	78 (53.4)	86 (52.4)	82 (54.3)	75 (47.2)
Jun–Oct (rainy)	*N* (%)	68 (46.6)	78 (47.6)	69 (45.7)	84 (52.8)
Maternal age (years) (*N* = 1 missing)	Median (IQR)	30.0 (25.1, 35.0)	29.8 (24.1, 33.7)	29.8 (24.0, 33.4)	29.5 (24.3, 34.2)
Maternal weight (kg) (*N* = 1 missing)	Mean (SD)	54.5 (7.7)	55.0 (9.7)	55.6 (8.7)	55.5 (9.8)
Maternal height (cm) (*N* = 2 missing)	Mean (SD)	161.9 (6.0)	162.2 (5.6)	162.1 (5.8)	161.9 (5.8)
Maternal BMI (kg/m^2^) (*N* = 3 missing)	Median (IQR)	20.6 (18.8, 22.4)	20.3 (18.7, 22.2)	20.5 (19.0, 22.5)	20.6 (19.2, 22.2)
Parity (*N* = 10 missing)					
0	*N* (%)	14 (9.7)	18 (11.2)	14 (9.4)	12 (7.7)
1–3	*N* (%)	39 (26.9)	56 (34.8)	54 (36.2)	52 (33.6)
4–12	*N* (%)	92 (63.5)	87 (54.0)	81 (54.4)	91 (58.7)

BMI = body mass index; FeFol = iron and folic acid; IQR = inter‐quartile range; LNS = lipid‐based nutritional supplement; MMN = multiple‐micronutrient; PE = protein‐energy; SD = standard deviation.

### 3.1. Primary outcomes

The key findings are summarized in Table 2. There was limited evidence that the supplements had affected fetal growth by 20 weeks of gestation, with the exception that fetal biometry measures were consistently greater in the PE arm compared to the other arms, with group‐wise comparisons for FL and AC being significant (e.g., FL *z*‐scores: FeFol −0.29, MMN −0.28, PE +0.13, PE + MMN −0.22; group‐wise *p* = .012). A similar pattern was observed at 30 weeks of gestation, but for different biometry measures (e.g., HC *z*‐scores: FeFol −0.42, MMN −0.58, PE −0.18, PE + MMN −0.39; group‐wise *p* = .010). Despite this evidence of between‐arm differences in some fetal biometry measures, likely due to higher values in the PE group, neonatal anthropometry *z*‐scores were not greater in the intervention arms than the FeFol arm (e.g., birth weight *z*‐scores: FeFol −0.71, MMN −0.63, PE −0.64, PE + MMN −0.62; group‐wise *p* = .796).

**Table 2:** Between‐intervention arm differences in primary and secondary outcomes

		Tablets		LNS		*P*‐values for between‐arm differences^a^
		FeFol	MMN	PE	PE + MMN	
		*N* = 146	*N* = 164	*N* = 151	*N* = 159	
Primary outcomes						
20 weeks gestation visit						
Femur length *z*‐score^b^ (*N* = 10 missing)	Mean (SD)	−0.29 (1.25)	−0.28 (1.27)	0.13 (1.29)	−0.22 (1.26)	.012
Head circumference z‐score^b^ (*N* = 8 missing)	Mean (SD)	−0.07 (1.20)	−0.02 (1.18)	0.20 (1.12)	0.04 (1.15)	.222
Bi‐parietal diameter *z*‐score^b^ (*N* = 8 missing)	Mean (SD)	−0.96 (1.08)	−0.91 (0.95)	−0.74 (0.99)	−0.83 (0.97)	.244
Occipital‐frontal diameter *z*‐score^b^ (*N* = 8 missing)	Mean (SD)	−0.13 (1.30)	−0.09 (1.31)	0.14 (1.31)	−0.03 (1.32)	.316
Abdominal circumference *z*‐score^b^ (*N* = 9 missing)	Mean (SD)	−0.54 (1.20)	−0.68 (1.15)	−0.30 (1.17)	−0.39 (1.19)	.024
30 weeks gestation visit						
Femur length *z*‐score^b^ (*N* = 10 missing)	Mean (SD)	0.32 (1.20)	0.25 (1.25)	0.42 (1.24)	0.33 (1.19)	.662
Head circumference *z*‐score^b^ (*N* = 7 missing)	Mean (SD)	−0.42 (1.13)	−0.58 (0.96)	−0.18 (1.03)	−0.39 (1.04)	.010
Bi‐parietal diameter *z*‐score^b^ (*N* = 4 missing)	Mean (SD)	−1.04 (0.98)	−1.09 (0.86)	−0.82 (0.87)	−0.94 (0.89)	.044
Occipital‐frontal diameter *z*‐score^b^ (*N* = 5 missing)	Mean (SD)	−0.39 (1.06)	−0.39 (1.00)	−0.28 (1.07)	−0.41 (1.10)	.689
Abdominal circumference *z*‐score^b^ (*N* = 7 missing)	Mean (SD)	0.01 (1.15)	−0.11 (1.06)	0.18 (1.09)	0.05 (1.01)	.123
Birth visit						
Weight *z*‐score^c^	Mean (SD)	−0.71 (0.84)	−0.63 (0.94)	−0.64 (0.87)	−0.62 (0.86)	.796
Length *z*‐score^c^ (*N* = 2 missing)	Mean (SD)	−0.16 (0.90)	−0.15 (1.03)	−0.23 (0.96)	−0.05 (0.90)	.418
Head circumference *z*‐score^c^ (*N* = 4 missing)	Mean (SD)	−0.84 (1.08)	−0.86 (1.11)	−0.94 (0.99)	−0.83 (1.18)	.817
Weight‐for‐length *z*‐score^c^ (*N* = 11 missing)	Mean (SD)	−1.05 (1.26)	−0.90 (1.34)	−0.86 (1.28)	−1.03 (1.33)	.481
Secondary outcomes						
Birth visit						
Gestational age (weeks)	Median (IQR)	40.1 (39.2, 41.1)	40.4 (39.5, 41.2)	39.8 (39.1, 40.7)	40.3 (39.4, 41.1)	.016
Preterm (<37 weeks gestation)	*N* (%)	4 (2.7)	3 (1.8)	2 (1.3)	4 (2.5)	.819
LBW (<2.5 kg)	*N* (%)	18 (12.3)	16 (9.8)	13 (8.6)	15 (9.4)	.736
SGA (<10th percentile^b^)	*N* (%)	52 (35.6)	52 (31.7)	55 (36.4)	51 (32.1)	.751
Maternal weight gain (kg/week)						
Booking to the 20 week visit (*N* = 4 missing)	Mean (SD)	0.34 (0.31)	0.39 (0.33)	0.41 (0.35)	0.36 (0.40)	.289
Booking to the 30 week visit (*N* = 3 missing)	Mean (SD)	0.32 (0.16)	0.33 (0.18)	0.32 (0.17)	0.32 (0.19)	.826
20 week visit to the 30 week visit (*N* = 6 missing)	Mean (SD)	0.30 (0.19)	0.30 (0.23)	0.28 (0.19)	0.31 (0.21)	.799

ANOVA = analysis of variance; BMI = body mass index; FeFol = iron and folic acid; IQR = inter‐quartile range; LNS = lipid‐based nutritional supplement; LBW = low birth weight; MMN = multiple‐micronutrient; PE = protein‐energy; SGA = small‐for‐gestational age; SD = standard deviation; WHO = World Health Organization.

a Between‐arm differences were tested using ANOVA or Kruskal–Wallis for continuous variables and Chi‐squared tests for categorical variables.

b *z*‐scores and percentiles according to the INTERGROWTH‐21st standards.

c *z*‐scores according to the WHO growth standards.

### 3.2. Secondary outcomes

On average, births occurred at an earlier gestational age in the PE arm compared to the other arms (FeFol 40.1, MMN 40.4, PE 39.8, PE + MMN 40.3; group‐wise *p* = .016), although were no between‐arm differences in rates of preterm, low birth weight, and SGA (Table 2). There were also no between‐arm differences in maternal weight gain, which suggests that the nutritional supplements were not being utilized by the mother herself for greater gestational weight gain.

### 3.3. Association modification

Table 3 presents intervention‐by‐potential modifier estimates from 56 separate models. There was no evidence that maternal height or BMI at booking modified any of the intervention associations with the outcomes, but there was evidence that some of the interventions were less efficacious in the wet season compared to the dry season for some outcomes at 30 weeks gestation. For example, the association of MMN (relative to FeFol) with OFD was 0.482 (95% CI [−0.955, −0.009]) *z*‐scores lower if assessment occurred in the wet season compared to the dry season. Accordingly, in the stratified analyses presented in Table 4, estimates were generally larger/positive for the sub‐group measured in the dry season and smaller/negative for the sub‐group measured in the rainy season. Similarly, some intervention‐by‐maternal weight gain terms were positive and significant in the neonatal anthropometry models in Table 3, suggesting that the interventions were more efficacious among women with greater gestational weight gain. Indeed, in the stratified analyses presented in Table 4, all of the supplements had significant positive associations with birth weight *z*‐scores among mothers who were in the highest quartile of maternal weight gain (e.g., PE + MMN estimate = + 0.462; 0.097, 0.826) but not among those who were in the lowest quartile (e.g., PE + MMN estimate = −0.099; −0.459, 0.260). Similar evidence of association modification by maternal weight gain was observed for HC and WFL, but not length *z*‐scores.

**Table 3:** General linear regression models^a^ testing for modification of intervention associations with outcomes

Interaction tested	Outcome	*N*	Interaction estimates		
			MMN	PE	PE + MMN
			B [95% CI] P	B [95% CI] P	B [95% CI] P
Intervention X season of measurement (0 = dry, 1 = wet)	20 weeks gestation visit				
	Femur length *z*‐score^b^	610	0.479 [−0.093, 1.052] 0.101	0.350 [−0.234, 0.934] 0.239	−0.076 [−0.653, 0.502] 0.797
	Head circumference *z*‐score^b^	612	0.215 [−0.309, 0.739] 0.421	−0.190 [−0.726, 0.346] 0.487	−0.288 [−0.817, 0.242] 0.287
	Bi‐parietal diameter *z*‐score^b^	612	0.009 [−0.441, 0.459] 0.968	0.115 [−0.345, 0.575] 0.624	0.030 [−0.425, 0.485] 0.898
	Occipital‐frontal diameter *z*‐score^b^	612	0.208 [−0.382, 0.798] 0.489	0.101 [−0.503, 0.704] 0.743	−0.429 [−1.026, 0.167] 0.158
	Abdominal circumference *z*‐score^b^	611	0.411 [−0.121, 0.943] 0.130	0.084 [−0.460, 0.627] 0.762	−0.092 [−0.630, 0.445] 0.736
	30 weeks gestation visit				
	Femur length *z*‐score^b^	610	−0.502 [−1.052, 0.049] 0.074	−0.264 [−0.827, 0.298] 0.357	−0.043 [−0.597, 0.511] 0.879
	Head circumference *z*‐score^b^	613	−0.441 [−0.908, 0.025] 0.064	−0.385 [−0.862, 0.091] 0.113	−0.264 [−0.734, 0.206] 0.271
	Bi‐parietal diameter *z*‐score^b^	616	−0.387 [−0.790, 0.016] 0.060	−0.109 [−0.520, 0.303] 0.604	−0.138 [−0.544, 0.269] 0.506
	Occipital‐frontal diameter *z*‐score^b^	615	−0.482 [−0.955, −0.009] 0.046	−0.731 [−1.213, −0.249] 0.003	−0.309 [−0.785, 0.168] 0.204
	Abdominal circumference *z*‐score^b^	613	−0.235 [−0.717, 0.248] 0.339	−0.554 [−1.048, −0.060] 0.028	−0.040 [−0.527, 0.446] 0.871
	Birth visit				
	Weight *z*‐score^c^	620	−0.140 [−0.543, 0.263] 0.496	−0.186 [−0.591, 0.220] 0.369	−0.169 [−0.577, 0.238] 0.415
	Length *z*‐score^c^	618	−0.331 [−0.769, 0.107] 0.138	−0.369 [−0.809, 0.070] 0.099	−0.093 [−0.534, 0.348] 0.680
	Head circumference *z*‐score^c^	616	−0.136 [−0.642, 0.369] 0.597	−0.114 [−0.622, 0.394] 0.660	−0.130 [−0.641, 0.380] 0.617
	Weight‐for‐length *z*‐score^c^	609	0.072 [−0.538, 0.682] 0.817	−0.004 [−0.611, 0.604] 0.990	−0.238 [−0.848, 0.372] 0.443
Intervention X maternal height at booking (cm)	20 weeks gestation visit				
	Femur length *z*‐score^b^	608	−0.006 [−0.055, 0.043] 0.811	−0.019 [−0.068, 0.030] 0.449	−0.008 [−0.056, 0.041] 0.748
	Head circumference *z*‐score^b^	610	−0.008 [−0.052, 0.037] 0.739	−0.016 [−0.061, 0.029] 0.484	−0.029 [−0.073, 0.016] 0.202
	Bi‐parietal diameter *z*‐score^b^	610	−0.003 [−0.041, 0.036] 0.891	−0.008 [−0.046, 0.031] 0.702	−0.020 [−0.058, 0.018] 0.306
	Occipital‐frontal diameter *z*‐score^b^	610	−0.021 [−0.072, 0.029] 0.406	−0.015 [−0.066, 0.035] 0.551	−0.022 [−0.072, 0.028] 0.394
	Abdominal circumference *z*‐score^b^	609	−0.018 [−0.063, 0.028] 0.451	−0.016 [−0.062, 0.030] 0.491	−0.027 [−0.072, 0.018] 0.237
	30 weeks gestation visit				
	Femur length *z*‐score^b^	608	−0.010 [−0.057, 0.037] 0.663	−0.012 [−0.059, 0.035] 0.622	0.014 [−0.033, 0.060] 0.559
	Head circumference *z*‐score^b^	611	−0.010 [−0.050, 0.031] 0.639	−0.016 [−0.056, 0.025] 0.442	−0.021 [−0.061, 0.018] 0.292
	Bi‐parietal diameter *z*‐score^b^	614	−0.003 [−0.038, 0.032] 0.859	−0.018 [−0.053, 0.017] 0.321	−0.010 [−0.044, 0.024] 0.571
	Occipital‐frontal diameter *z*‐score^b^	613	−0.004 [−0.045, 0.037] 0.856	0.000 [−0.041, 0.041] 0.992	−0.002 [−0.043, 0.038] 0.906
	Abdominal circumference *z*‐score^b^	611	−0.013 [−0.055, 0.029] 0.536	−0.010 [−0.052, 0.032] 0.637	−0.019 [−0.060, 0.023] 0.376
	Birth visit				
	Weight *z*‐score^c^	618	0.009 [−0.024, 0.043] 0.584	0.017 [−0.017, 0.051] 0.325	0.013 [−0.020, 0.046] 0.449
	Length *z*‐score^c^	616	0.011 [−0.025, 0.047] 0.558	0.023 [−0.013, 0.060] 0.206	0.020 [−0.015, 0.056] 0.267
	Head circumference *z*‐score^c^	614	0.021 [−0.022, 0.063] 0.335	0.016 [−0.026, 0.059] 0.453	0.019 [−0.023, 0.061] 0.367
	Weight‐for‐length *z*‐score^c^	607	0.004 [−0.047, 0.055] 0.880	−0.002 [−0.053, 0.049] 0.931	−0.004 [−0.054, 0.046] 0.875
Intervention X maternal BMI at booking (kg/m^2^)	20 weeks gestation visit				
	Femur length *z*‐score^b^	607	0.000 [−0.101, 0.100] 0.994	0.022 [−0.084, 0.128] 0.683	0.019 [−0.083, 0.121] 0.711
	Head circumference *z*‐score^b^	609	−0.011 [−0.102, 0.081] 0.819	0.029 [−0.068, 0.127] 0.555	0.023 [−0.071, 0.116] 0.634
	Bi‐parietal diameter *z*‐score^b^	609	−0.018 [−0.097, 0.060] 0.650	0.004 [−0.079, 0.087] 0.926	0.012 [−0.068, 0.093] 0.761
	Occipital‐frontal diameter *z*‐score^b^	609	0.022 [−0.081, 0.125] 0.677	0.025 [−0.085, 0.135] 0.657	0.026 [−0.080, 0.131] 0.635
	Abdominal circumference *z*‐score^b^	608	−0.088 [−0.181, 0.005] 0.063	−0.064 [−0.163, 0.034] 0.200	−0.080 [−0.175, 0.015] 0.097
	30 weeks gestation visit				
	Femur length *z*‐score^b^	607	0.029 [−0.067, 0.125] 0.555	0.036 [−0.066, 0.138] 0.491	−0.025 [−0.123, 0.074] 0.622
	Head circumference *z*‐score^b^	610	−0.010 [−0.092, 0.071] 0.803	0.012 [−0.074, 0.098] 0.787	−0.040 [−0.123, 0.043] 0.346
	Bi‐parietal diameter *z*‐score^b^	613	0.027 [−0.042, 0.096] 0.443	−0.018 [−0.092, 0.056] 0.629	−0.048 [−0.119, 0.023] 0.183
	Occipital‐frontal diameter *z*‐score^b^	612	0.045 [−0.036, 0.126] 0.278	0.034 [−0.053, 0.121] 0.444	−0.004 [−0.088, 0.080] 0.925
	Abdominal circumference *z*‐score^b^	610	0.049 [−0.033, 0.132] 0.243	0.035 [−0.053, 0.124] 0.433	−0.022 [−0.108, 0.063] 0.609
	Birth visit				
	Weight *z*‐score^c^	617	0.012 [−0.054, 0.078] 0.720	−0.015 [−0.086, 0.056] 0.671	−0.007 [−0.076, 0.061] 0.834
	Length *z*‐score^c^	615	−0.003 [−0.076, 0.070] 0.932	−0.001 [−0.080, 0.077] 0.974	0.000 [−0.076, 0.075] 0.991
	Head circumference *z*‐score^c^	613	−0.030 [−0.113, 0.053] 0.475	−0.069 [−0.159, 0.020] 0.129	−0.058 [−0.144, 0.029] 0.190
	Weight‐for‐length *z*‐score^c^	606	0.030 [−0.070, 0.130] 0.560	−0.037 [−0.144, 0.070] 0.496	−0.009 [−0.112, 0.094] 0.871
Intervention X maternal weight gain from booking (kg/week)^d^	20 weeks gestation visit				
	Femur length *z*‐score^b^	608	−0.225 [−1.129, 0.678] 0.624	0.011 [−0.888, 0.910] 0.980	−0.394 [−1.231, 0.443] 0.355
	Head circumference *z*‐score^b^	610	0.298 [−0.526, 1.122] 0.478	0.365 [−0.457, 1.186] 0.384	−0.045 [−0.809, 0.720] 0.909
	Bi‐parietal diameter *z*‐score^b^	610	0.475 [−0.229, 1.179] 0.185	0.542 [−0.159, 1.243] 0.130	−0.098 [−0.751, 0.555] 0.768
	Occipital‐frontal diameter *z*‐score^b^	610	0.394 [−0.536, 1.323] 0.406	0.506 [−0.421, 1.432] 0.284	0.037 [−0.825, 0.899] 0.933
	Abdominal circumference *z*‐score^b^	609	−0.215 [−1.057, 0.627] 0.616	0.368 [−0.471, 1.206] 0.389	−0.141 [−0.921, 0.640] 0.724
	30 weeks gestation visit				
	Femur length *z*‐score^b^	609	−0.573 [−2.206, 1.060] 0.491	−1.638 [−3.330, 0.053] 0.058	−0.279 [−1.853, 1.295] 0.728
	Head circumference *z*‐score^b^	612	−0.338 [−1.727, 1.051] 0.633	0.059 [−1.381, 1.499] 0.936	0.405 [−0.935, 1.745] 0.553
	Bi‐parietal diameter *z*‐score^b^	615	−0.489 [−1.687, 0.709] 0.423	0.229 [−1.014, 1.471] 0.718	−0.010 [−1.167, 1.147] 0.986
	Occipital‐frontal diameter *z*‐score^b^	614	−0.169 [−1.592, 1.255] 0.816	−0.003 [−1.472, 1.465] 0.996	0.375 [−0.993, 1.743] 0.590
	Abdominal circumference *z*‐score^b^	612	−0.149 [−1.589, 1.292] 0.839	−0.086 [−1.579, 1.408] 0.910	−0.510 [−1.902, 0.881] 0.471
	Birth visit				
	Weight *z*‐score^c^	617	0.349 [−0.809, 1.507] 0.554	0.759 [−0.443, 1.961] 0.215	0.410 [−0.709, 1.530] 0.472
	Length *z*‐score^c^	615	−1.075 [−2.331, 0.182] 0.094	−0.610 [−1.914, 0.694] 0.359	−0.465 [−1.680, 0.749] 0.452
	Head circumference *z*‐score^c^	613	1.097 [−0.361, 2.555] 0.140	1.692 [0.181, 3.202] 0.028	0.950 [−0.457, 2.358] 0.185
	Weight‐for‐length *z*‐score^c^	606	1.807 [0.062, 3.552] 0.042	2.029 [0.228, 3.829] 0.027	1.303 [−0.373, 2.980] 0.127

BMI = body mass index; FeFol = iron and folic acid; MMN = multiple‐micronutrient; PE = protein‐energy; WHO = World Health Organization.

a Each row represents a separate model including the interaction term and its components. The interaction estimates represent the change in the intervention association with the outcome (relative to the FeFol arm) for each unit increase in the potential modifier.

b *z*‐scores according to the INTERGROWTH‐21st standards.

c *z*‐scores according to the WHO growth standards.

d Maternal weight gain from booking to the 20 week visit for the 20 week outcomes, and from booking to the 30 week visit for the 30 week and birth outcomes.

**Table 4:** General linear regression models^a^ testing intervention associations with outcomes, stratified according to modifiers

	*N*	MMN	PE	PE + MMN
		B [95% CI] P	B [95% CI] P	B [95% CI] P
30 weeks gestation visit				
Femur length *z*‐score^b^				
If season of measurement Nov–May (dry)	307	0.230 [−0.075, 0.535] 0.139	0.109 [−0.210, 0.429] 0.501	−0.031 [−0.335, 0.272] 0.840
If season of measurement Jun–Oct (rainy)	299	−0.384 [−0.713, −0.055] 0.022	−0.001 [−0.330, 0.328] 0.997	−0.147 [−0.491, 0.197] 0.402
Head circumference *z*‐score^b^				
If season of measurement Nov–May (dry)	312	0.083 [−0.199, 0.364] 0.563	0.343 [0.050, 0.636] 0.022	0.112 [−0.167, 0.392] 0.429
If season of measurement Jun–Oct (rainy)	297	−0.375 [−0.701, −0.049] 0.024	0.049 [−0.277, 0.375] 0.769	−0.104 [−0.444, 0.236] 0.547
Bi‐parietal diameter *z*‐score^b^				
If season of measurement Nov–May (dry)	313	0.089 [−0.155, 0.333] 0.472	0.187 [−0.068, 0.441] 0.149	0.116 [−0.126, 0.359] 0.346
If season of measurement Jun–Oct (rainy)	299	−0.234 [−0.511, 0.044] 0.098	0.161 [−0.116, 0.439] 0.253	0.026 [−0.264, 0.316] 0.860
Occipital‐frontal diameter *z*‐score^b^				
If season of measurement Nov–May (dry)	312	0.239 [−0.038, 0.517] 0.091	0.370 [0.082, 0.659] 0.012	0.059 [−0.217, 0.334] 0.675
If season of measurement Jun–Oct (rainy)	299	−0.270 [−0.571, 0.031] 0.079	−0.216 [−0.517, 0.085] 0.159	−0.222 [−0.537, 0.093] 0.167
Abdominal circumference *z*‐score^b^				
If season of measurement Nov–May (dry)	311	−0.038 [−0.311, 0.235] 0.786	0.342 [0.057, 0.628] 0.019	0.005 [−0.267, 0.277] 0.971
If season of measurement Jun–Oct (rainy)	298	−0.244 [−0.574, 0.087] 0.148	−0.124 [−0.456, 0.207] 0.461	0.007 [−0.339, 0.352] 0.970
Birth visit				
Weight *z*‐score^c^				
If maternal weight gain^d^ quartile 4	154	0.371 [0.033, 0.710] 0.032	0.492 [0.126, 0.857] 0.009	0.462 [0.097, 0.826] 0.013
If maternal weight gain^d^ quartiles 2 & 3	309	−0.099 [−0.364, 0.166] 0.463	0.071 [−0.187, 0.329] 0.588	−0.001 [−0.263, 0.260] 0.992
If maternal weight gain^d^ quartile 1	152	−0.097 [−0.474, 0.281] 0.613	−0.261 [−0.660, 0.137] 0.197	−0.099 [−0.459, 0.260] 0.585
Length *z*‐score^c^				
If maternal weight gain^d^ quartile 4	153	−0.168 [−0.558, 0.221] 0.394	−0.151 [−0.570, 0.268] 0.477	0.075 [−0.343, 0.493] 0.723
If maternal weight gain^d^ quartiles 2 & 3	308	−0.167 [−0.473, 0.139] 0.283	−0.082 [−0.381, 0.216] 0.587	0.146 [−0.156, 0.447] 0.343
If maternal weight gain^d^ quartile 1	152	0.448 [0.045, 0.852] 0.030	0.189 [−0.238, 0.616] 0.384	0.108 [−0.276, 0.493] 0.579
Head circumference *z*‐score^c^				
If maternal weight gain^d^ quartile 4	153	0.297 [−0.129, 0.722] 0.170	0.442 [−0.017, 0.902] 0.059	0.520 [0.061, 0.979] 0.027
If maternal weight gain^d^ quartiles 2 & 3	307	−0.193 [−0.537, 0.151] 0.271	−0.047 [−0.382, 0.288] 0.784	−0.089 [−0.428, 0.249] 0.603
If maternal weight gain^d^ quartile 1	151	−0.088 [−0.574, 0.398] 0.721	−0.582 [−1.093, −0.072] 0.026	−0.149 [−0.609, 0.311] 0.524
Weight‐for‐length *z*‐score^c^				
If maternal weight gain^d^ quartile 4	152	0.855 [0.245, 1.465] 0.006	1.129 [0.476, 1.783] 0.001	0.772 [0.121, 1.424] 0.021
If maternal weight gain^d^ quartiles 2 & 3	302	0.064 [−0.336, 0.465] 0.753	0.171 [−0.217, 0.558] 0.387	−0.195 [−0.586, 0.195] 0.326
If maternal weight gain^d^ quartile 1	150	−0.577 [−1.230, 0.076] 0.083	−0.588 [−1.274, 0.097] 0.092	−0.272 [−0.889, 0.344] 0.384

BMI = body mass index; FeFol = iron and folic acid; MMN = multiple‐micronutrient; PE = protein‐energy; WHO = World Health Organization.

a Each row represents a separate model, and the estimates represent the association of each intervention with the outcome (relative to the FeFol arm). The models for outcomes at the 30 weeks gestation visit were adjusted for sex, gestational age, gestational age at booking, sonographer, and maternal height at booking, BMI at booking, and weight gain from booking to the 30 week visit. The models for outcomes at the birth visit were adjusted for sex, parity, gestational age, age at measurement, season of measurement, and maternal height at booking, BMI at booking, and weight gain from booking to the 30 week visit.

b *z*‐scores according to the INTERGROWTH‐21st standards.

c *z*‐scores according to the WHO growth standards.

d Maternal weight gain from booking to 30 week visit.

Nearly identical results to those presented in this paper were obtained (a) in analyses restricted to individuals in the top three quartiles of compliance (standardized within each arm) or length of time on supplement and (b) in analyses using neonatal anthropometry from a week one visit instead of the birth visit (data not presented).

## 4 DISCUSSION

This study conducted a post‐hoc analysis in a prenatal trial of nutritional supplementation in rural Gambia. Despite evidence of between‐arm differences in some fetal biometry measures, likely due to higher values in the PE group, *z*‐scores at birth were not greater in the intervention arms than the FeFol arm. Our key finding, therefore, is that prenatal LNS intervention was not associated with better fetal growth in the whole sample. Pronounced seasonality in The Gambia affects many aspects of diet, health, and behavior (Moore, 2016), thereby providing a robust design to explore how efficacy within a single population might vary depending on nutritional status. In sub‐group analyses, evidence was found to suggest that the supplements were more efficacious in the dry season than the rainy season (for some fetal biometry outcomes at 30 weeks of gestation) and among mothers who demonstrated the greatest gestational weight gain (for some neonatal anthropometry outcomes). These results, however, need to be interpreted with caution given that the study was not prospectively designed to test for such association modification.

The finding that both PE and/or MMN supplementation were significantly associated with increased birth weight, albeit only in a sub‐group, is in agreement with the most recent Cochrane reviews (Haider & Bhutta, 2015; Ota et al., 2015). Existing evidence also supports our finding that MMN supplementation was associated with increased birth weight only in well‐nourished women (as indicated by better gestational weight gain; Haider & Bhutta, 2015; Fall, Fisher, Osmond, Maternal, & Micronutrient, 2009). However, the existing evidence showing that PE supplementation is more effective in undernourished women is opposite to our finding that PE supplementation was associated with increased birth weight only in well‐nourished women (as indicated by better gestational weight gain; Imdad & Bhutta, 2012). It may be that the sub‐group of women in the present study, in which significant associations were observed, was macronutrient deficient (thereby increasing the efficacy of PE supplementation) but not micronutrient deficient (thereby increasing the efficacy of MMN supplementation). This proposition ties in somewhat with the observation that women in the highest quartile of gestational weight gain actually weighed about 6 kg less at booking than women in the lowest quartile, and it was not until 30 weeks of gestation that the two groups weighed approximately the same (59.4 vs. 60.8 kg).

LNS products are a relatively recent development, and as such, few prenatal trials have been completed and published. The results so far have been equivocal with no evidence of a positive effect on birth size in Malawi (Ashorn et al., 2015), some evidence among younger women and those with household food insecurity in Bangladesh (Mridha et al., 2016), some evidence among primiparous women in Ghana (Adu‐Afarwuah et al., 2015), and some evidence in Burkina Faso but only for length and only among births occurring at the end of the rainy season (Huybregts et al., 2009; Toe et al., 2015). The sub‐group association of LNS supplementation with birth weight in the present study but not in Burkina Faso could be explained by the higher daily energy dose provided by the LNS product used in The Gambia (746 vs. 372 kcal/day) and by the fact that the control group in Burkina Faso also received MMN, which are known to increase birth weight. The association of LNS supplementation with birth length in Burkina Faso but not in The Gambia was surprising, particularly given that we supplemented at two times the UNICEF/WHO/United Nations University formulation of key micronutrients, while in Burkina Faso, they only supplemented at one times the formulation. It may be that the Gambians were more micronutrient deficient or that the Burkinabes had a lower threshold for the intervention to work because stunting is more prevalent in Burkina Faso than The Gambia (UNICEF‐WHO‐The World Bank, 2015). For policy‐makers, such inconsistent results between trials means that the scale‐up of antenatal LNS‐based supplements for SGA rate reduction may not yet be justified.

The ability to gain weight during gestation is seasonally patterned in The Gambia (Poppitt et al., 1994), so it makes sense that we observed association modification by both season and gestational weight gain. Taken together, our interpretation of the association modification results is that environments conducive to better gestational weight gain (i.e., the dry season) may allow the mother to be more nutritionally replete, such that any additional nutrients from supplementation can be used to support fetal growth and development. This reflects a classic trade‐off scenario between optimization of maternal nutritional status and fetal growth and development that has been reported on previously (Rasmussen & Habicht, 2010; Lechtig, Yarbrough, Delgado, Habicht, & Klein, 1975). Previous trials in The Gambia and other countries with marked seasonality have found stronger positive effects of supplementation for births occurring in the nutritionally debilitating rainy season (Toe et al., 2015; Ceesay et al., 1997). This finding, however, is not mutually exclusive from the findings of the present study as 43% of women in the highest quartile of gestational weight gain (in whom the intervention seemed to work) actually went on to deliver in the rainy season, compared to just 25% of women in the lowest quartile of gestational weight gain.

One unexpected finding was that differences in fetal biometry were likely due to higher values in the PE arm compared to the PE + MMN arm. This was more likely due to baseline differences in fetal size at booking (BPD 30.2 vs. 28.5 mm) persisting to 20 and 30 weeks of gestation than differential compliance between the two LNS arms (81.7% vs. 81.2%, respectively). The fetal biometry results, therefore, have to be interpreted with caution, particularly given that we did not observe any between‐arm differences in weight, length, or HC at birth in the whole sample.

The main strengths of the present study are that (a) supplementation started early in gestation (~13 weeks), which is important knowing that even pre‐conceptional nutritional status may impact on birth weight (Potdar et al., 2014; Young et al., 2015); and that (b) fetal biometry as well as neonatal anthropometry measures were available and analyzed, thereby providing us with the opportunity to investigate the ages in development when the supplements might have first started to affect growth. In terms of limitations, this was a post‐hoc analysis with a sample size that was not powered on the outcomes presented in this paper or for the sub‐group analyses. Average power to detect an intervention association with an outcome in our final regression models (Table 4) was 0.48, so we had a relatively high risk of rejecting false null hypotheses (i.e., type two errors). The fact that we generally found consistent association modification across the different measures (e.g., birth weight, HC, and WFL) by variables that we know are related to each other in The Gambia (i.e., season and gestational weight gain) suggests, however, that these key findings are not chance. Nonetheless, further research is needed to confirm our findings and reveal which components or casual factors of maternal weight gain may increase the efficacy of prenatal nutritional intervention. Other limitations include not having a maternal weight measure at the very end of gestation, which would have allowed us to quantify weight gain throughout the final trimester, and not necessarily being able to generalize our results to other populations.

In conclusion, the present paper found no strong evidence that a prenatal LNS intervention was associated with better fetal growth in the whole sample. Sub‐group analyses did, however, reveal positive and significant associations of all interventions (i.e., MMN, PE, or MMN + PE vs. FeFol) with birth weight and HC among women who demonstrated the greatest gestational weight gain.

## SOURCE OF FUNDING

This trial is supported by the UK Medical Research Council (MRC) (MC‐A760‐5QX00) and the UK Department for International Development (DFID) under the MRC/DFID Concordat agreement. WJ and SEM are funded by the UK MRC programme MC_UP_1005/1.

## CONFLICT OF INTEREST

The authors declare that they have no conflicts of interest.

## CONTRIBUTIONS

The authors' responsibilities were as follows: SEM, MKD, and AMP conceived and designed the ENID Trial; MKD, FS, PN, and SEM conducted the research; WJ and SEM conceived this post‐hoc analysis; WJ performed the statistical analyses and drafted the manuscript. All authors critically revised and approved the manuscript.
